# Supplementary material for: Effects of rumen-protected or unprotected Clostridium butyricum on growth performance, rumen fermentation, immunity and antioxidation in fattening goats
Source: PLoS One. 2025 Oct 10;20(10):e0330512. doi: 10.1371/journal.pone.0330512 (PMC12513589; doi:10.1371/journal.pone.0330512)
Supplement: Attachment — Submitted filename: Repose to Reviewer.docx [file pone.0330512.s002.docx]

Dear Reviewer:

We are truly grateful for your critical comments and thoughtful suggestions for our manuscript. These comments and suggestions were helpful, and we have revised the manuscript carefully. The revised portions have been marked in red in the revised manuscript. Our point-by-point responses to your comments/suggestions are provided below.

**Comment 1:** In abstract section, avoid redundancy, such as repeating "rumen-protected andunprotected C. butyricum" and statistical results (P values) should be included forall key findings mentioned in the abstract.

**Response 1:**

Thank you for your suggestion. I apologize for the unnecessary misunderstanding caused by our mistakes. On the basis of the suggestions and guidance from you and the other reviewers, we have modified the repetitive expressions and supplemented the statistical results in the revised manuscript. **(Lines 11--32)**

**Comment 2:** In introduction section, please providePotential mechanisms through which C. butyricum affects immune performanceand include more recent references from 2020-2024 to contextualize the study.

**Response 2:**

Thank you for your suggestion. The revised manuscript has been supplemented with information on the potential mechanism of action of *C. butyricum* in enhancing immune function and has updated the references. **(Lines 46--61)**

**Comment 3:** Furthermore, hypotheses should be clearly stated in introduction section and objectives should be based on strong hypotheses.

**Response 3:**

Thank you for your suggestion. We have rewritten the section in question. **(Lines 62--69)**

**Comment 4:** In result section, highlightspecific changes in microbial genera that are relevant to immunity or antioxidation.

**Response 4:**

Thank you for your suggestion. In the revised version, the specific changes in the microorganisms of mice related to immune function have been supplemented according to your suggestions. **(Lines 319--331)**

**Comment** **5:** For example, avoid claiming "better immune protection" without stronger statistical backing. Please also explain potential mechanisms for the observed effects (or lack thereof) of the treatments.

**Response 5:**

Thank you for your suggestion. According to your suggestions, the expressions of some conclusions have been changed in the revised manuscript. **(Lines 448--456)**

Six hours after the intraperitoneal injection of LPS and the collection of serum samples, the IgM level in the CB group was significantly greater than that in the other two groups. Currently, we do not have a very clear mechanism to explain this phenomenon. In subsequent research, we will focus on potential mechanisms in this regard.

**Comment 6:** In the last the legends of table and figure shouldprovide more detailed explanations, especially regarding abbreviations andstatistical notations. Please also include the number of replicates (N) in all figure legends.

**Response 6:**

Thank you for your suggestion. According to your suggestion, provide a more detailed explanation of the legends for both the tables and the figures, and indicate the number of repetitions (N) in all the legends. **(Lines 245--248, 298--303)**
