# Supplementary material for: Effects of rumen-protected or unprotected Clostridium butyricum on growth performance, rumen fermentation, immunity and antioxidation in fattening goats
Source: PLoS One. 2025 Oct 10;20(10):e0330512. doi: 10.1371/journal.pone.0330512 (PMC12513589; doi:10.1371/journal.pone.0330512)
Supplement: Attachment — Submitted filename: Response to Reviewers.docx [file pone.0330512.s003.docx]

Dear Reviewer:

We are truly grateful for your critical comments and thoughtful suggestions for our manuscript. These comments and suggestions were helpful, and we have revised the manuscript carefully. The revised portions have been marked in red in the revised manuscript. Our point-by-point responses to your comments/suggestions are provided below.

**Reviewer #2:**

**Comment 1:** The introduction needs to be improved, and English language editing might be required.

**Response 1:** Thank you for your suggestion. We have conducted a detailed and accurate English edit of the introduction section in accordance with your suggestions.

**Reviewer #3:**

**Comment 1:** Line 15: please correct rumen protected and rumen unprotected in sentence ‘…effects of rumen protection with or without C. butyricum’.

**Response 1:** Thank you for your suggestion. We have carefully revised the relevant sentences in accordance with your suggestions. (Line 15)

**Comment 2:** Line 23-24: these are results ‘thus alleviating the decrease in rumen pH’ not discussion please indicate it in the form of result.

**Response 2:** Thank you for your suggestion. We have carefully revised the relevant sentences in accordance with your suggestions. (Line 23-24)

**Comment 3:** Line 25: please remove it ‘albeit through different mechanisms’.

**Response 3:** Thank you for your suggestion. We have carefully revised the relevant sentences in accordance with your suggestions. (Line 25)

**Comment 4:** Line 25-27: please be specific ‘The difference in the fecal flora between the CB and RPCB groups was limited, but the content of harmful bacteria in the feces of the other two groups decreased compared with that in the CON group’ what do you mean by harmful bacteria.

**Response 4:** Thank you for your suggestion. ‘Harmful bacteria’ refer to those bacteria present in the intestinal tract of goats that have adverse effects on the health or physiological functions of the host. For instance, certain pathogenic strains such as Salmonella and Escherichia coli that may exist in the intestines can cause problems like intestinal infections and stunted growth in ruminants, thereby exerting negative impacts on livestock production. (Line 25-27)

**Comment 5:** Line 38-40: don’t write anything without reference ‘When used as feed additives, antibiotics have been shown to increase ruminant feed utilization and inhibit the growth of harmful bacteria by increasing immunity and preventing disease’ please include citation. Anas S. Dablool, Banan Atwah, Saad Alghamdi, Maha Abdullah Momenah, Ohud Saleh, Nada Alhazmi, Yasser S. Mostafa, Saad A. Alamri, Worood A.A. Alyoubi, Naheda M. Alshammari, Alaa S. Mohamed, Nadeen G. Mostafa and Belal A. Omar. Could Paenibacillus xylanexedens MS58 be an Ecofriendly Antibiotic in Poultry Production? Impacts on Performance, Blood Biochemistry, Gut Microbiota and Meat Quality.Pak Vet J, 2024, 44(2): 352-360. And Fatimah S. Alqahtani, Safia M.A. Bahshwan, Mada M. AL-Qurashi, Aminah Allohibi, Eman A. Beyari, Mashail A. Alghamdi, Roua S. Baty, Nawal Al-Hoshani, Anas S. Dablool, Fadwa Mohammed Alkhulaifi, Abdullateef A. Alshehri, Essam H. Ibrahim,0, Ahmed M. Saad, Nadeen G. Mostafa

Impact of Dietary Bacillus toyonensis M44 as an Antibiotic Alternative on Growth, Blood Biochemical Properties, Immunity, Gut Microbiota, and Meat Quality of IR Broilers. Pak Vet J, 2024, 44(3): 637-646.

**Response 5:** Thank you for your suggestion. We have added relevant content in accordance with your suggestions and marked the citations. (Line 38-40)

**Comment 6:** Line 40-43: ‘However, the widespread use of antibiotics in animal feed has raised concerns over the accumulation of drug residues in the environment and animal products, which in turn contributes to the development of antibiotic-resistant bacteria and poses a risk to human health [2]’ citation is outdated nearly 10 year back. Please include recent citation. The citation could be ‘Rida Haroon Durrani, Ali Ahmad Sheikh, Muhammad Humza, Salman Ashraf, Aleena Kokab, Tauqeer Mahmood and Muhammad Umar Zafar Khan. Evaluation of Antibiotic Resistance Profile and Multiple Antibiotic Resistance Index in Avian Adapted Salmonella enterica serovar Gallinarum Isolates. Pak Vet J, 2024, 44(4): 1349-1352’ and ‘Magnoli AP, Parada J, Luna MarÃ¬a J, Corti M, Escobar FM, FernÃ¡ndez C, Coniglio MV, Ortiz ME, Wittouck P, Watson S, Cristofolini LA and Cavaglieri L, 2024. Impact of probiotic Saccharomyces cerevisiae var. boulardii RC009 alone and in combination with a phytase in broiler chickens fed with antibiotic-free diets. Agrobiological Records 16: 1-10.’ And ‘Ezzat M, Hassanin AAI, Mahmoud AE, Ismail SM and El-Tarabili RM, 2023. Risk factors, antibiotic profile, and molecular detection of virulence and antibiotic resistance genes of enteric bacteria in diarrheic calves in Egypt. International Journal of Veterinary Science 12(2): 161-168.’ And ‘Bui MTL, Nguyen TT, Nguyen HC, Ly KLT and Nguyen TK, 2024. Antibiotic resistance and pathogenicity of Escherichia coli isolated from cattle raised in households in the Mekong Delta, Vietnam. International Journal of Veterinary Science 13(5): 730-736.’

**Response 6:** Thank you for your suggestion. We have attached the latest citations in accordance with your suggestions. (Line 40-43)

**Comment 7:** Line 43-44: rewrite the sentence ‘Given the significant impact of livestock food production on both human health and the environment, the identification of new feed additives to replace antibiotics and enhance feed efficiency has become crucial’ and new sentence could be ‘Given the significant impact of livestock food production on both human health and the environment, the new feed additives to replace antibiotics and enhance feed efficiency are being used in livestock industry’ and also include citations ‘Abdul Hannan, Xiaoxia Du, Bakhtawar Maqbool3 and Ahrar Khan. Nanoparticles as Potent Allies in Combating Antibiotic Resistance: A Promising Frontier in Antimicrobial Therapy. Pak Vet J, 2024, 44(4): 957-967’ and ‘Maha J. Balgoon and Amira M. Alghamdi. Biochemical Assessment of Boswellic Acid Enrich-Frankincense Extract and its Antioxidant, Antibacterial, Anticancer and Anti-inflammatory Potential in Ameliorating the Glycerol-Toxicity in Rats. Pak Vet J, 2024, 44(4): 1023-1032’ and ‘Mohamed A. Elzaiat, Abdelrahman S. Mandour, Mohamed A. H. Youssef, Hany A. Wafa, Salma M. Aljahdali, Amani Osman Shakak, Latifa Al Husnain, Mohammed A. Alqahtani, Mashail A. Alghamdi, Amani Omar Abuzaid, Tahani M. Alqahtani, Hawazen K. Al Gheffari, Nahla Alsayd Bouqellah, Rania M.Y. Heakel Biochemical and Molecular Characterization of Five Basil Cultivars Extract for Enhancing the Antioxidant, Antiviral, Anticancer, Antibacterial, and Antifungal Activities. Pak Vet J, 2024, 44(4): 1105-1119’ and ‘Rashid S, Alsayeqh AF, Akhtar T, Abbas RZ and Ashraf R, 2023. Probiotics: Alternative of antibiotics in poultry production. International Journal of Veterinary Science 12(1): 45-53.’

**Response 7:** Thank you for your suggestion. We have revised the relevant content in accordance with your suggestions and marked the citations. (Line 43-44)

**Comment 8:** Please also provides background on *C. butyricum* use in livestock, but the literature review is not comprehensive and citations are outdated.

**Response 8:** Thank you for your suggestion. We have added the background information on the use of *C. butyricum* in livestock, completed the literature review, and cited the latest references. (Line 53-58)

**Comment 9:** The hypothesis is vague and not well-integrated with objectives.

**Response 9:** Thank you for your suggestion. We have rewritten the hypotheses to ensure they are well-aligned with the objectives. (Line 75-81)

**Comment 10:** Don’t you think use of multiple post hoc tests (LSD and Duncan) increase Type I error? How could you justify it?

**Response 10:** Thank you for your suggestion. We conducted post hoc comparisons using both LSD (for sensitivity) and Duncan’s test (for mean clustering). Since these tests do not strictly control family-wise error, we confirmed findings with Tukey’s HSD. Only results consistent across methods were interpreted as significant. (Line 189-192)

**Comment 11:** Overall, this section is good, however, the authors could explain why they attempt to draw performance implications while the data is non significant. This is misleading, please correct it. In microbial composition, diversity differences are minor and reported genus-level changes (e.g., Haemophilus, Flavonifractor) lack functional interpretation and are not linked convincingly to immunity. While in immune and antioxidant markers results only IgM shows significance post-LPS injection. Other indices (IgA, IgG, T-AOC, SOD, CAT, MDA) show no changes, yet the manuscript still draws generalized conclusions, please correct it in the revised manuscirpt. I would also comment that metabolomics results contain rich data, but poorly integrated into discussion. Identified changes in plasma metabolites are not contextualized in terms of relevance to goat health or productivity. Please correct grammar and support your study with strong data while in discussion for example several statements (e.g., improvement in immune response, metabolic shifts) are unsupported by robust data and the metabolic pathway discussion lacks depth and does not explain why those pathways are relevant for goats.

**Response 11:** Thank you for your suggestion. In our previous conclusion, we stated that Clostridium butyricum had no significant effect on growth performance and microbial composition. However, this might have been a misrepresentation due to incorrect English expression. We have now removed this content and only elaborated on the conclusion that Clostridium butyricum can enhance immune performance and promote the function of hepatic fatty acid metabolism. (Line 463-469)
